# Childhood Maltreatment, Low Serum Cortisol Levels, and Non-Suicidal Self-Injury in Young Adults With Major Depressive Disorders

**DOI:** 10.3389/fped.2022.822046

**Published:** 2022-06-03

**Authors:** Bo Peng, Jinmeng Li, Haitao Liu, Han Fang, Weitan Zhao, Guanjie Chen, Meihong Xiu, Yingli Zhang

**Affiliations:** ^1^Department of Depressive Disorders, Shenzhen Kangning Hospital, Shenzhen, China; ^2^Shenzhen Mental Health Center, Shenzhen, China; ^3^Department of Hematology, Peking University Shenzhen Hospital, Shenzhen, China; ^4^Shenzhen Longgang District Maternity & Child Healthcare Hospital, Shenzhen, China; ^5^Peking University HuiLongGuan Clinical Medical School, Beijing HuiLongGuan Hospital, Beijing, China

**Keywords:** depression, non-suicidal self-injury, childhood adversity, cortisol, association

## Abstract

**Background:**

Non-suicidal self-injury (NSSI) is a serious public health problem widely present among young adults and adolescents. While finding risk factors associated with NSSI among young patients with major depressive disorder (MDD) is challenging, the current study aims to measure childhood adversity and serum cortisol levels and elucidate their relationship in MDD patients with NSSI.

**Methods:**

In this cross-sectional study, 126 young patients with MDD (aged 16 to 35 years) were recruited. The depressive symptoms were assessed by the Beck Depression Inventory (BDI-II), and the childhood adversity was evaluated by the Chinese version of the Childhood Trauma Questionnaire (CTQ) scale. Serum cortisol levels were determined by the kits in patients.

**Results:**

Relative to MDD patients without NSSI, MDD patients with NSSI had a higher CTQ total score and its four subscores. Moreover, the cortisol levels in patients with MDD/NSSI were significantly decreased than in MDD patients without NSSI. For patients with MDD/NSSI, there is a negative association between cortisol levels and emotional neglect, but not for MDD patients without NSSI. Further regression analysis showed that low cortisol levels, BDI-II, and emotional neglect were risk factors for NSSI in young patients with MDD.

**Conclusion:**

Our findings suggest that young MDD patients with NSSI experience more childhood adversity and have lower cortisol levels. Also, lower cortisol levels were associated with childhood adversity but not with depressive symptoms. Further, lower cortisol levels, depressive symptoms, and emotional neglect were risk factors for NSSI in young patients with MDD.

## Introduction

Non-suicidal self-injury (NSSI) refers to the intention and repeated action to injure their bodies without suicidal intention and taking a series of direct actions (1). It is more common in the early stages of adults and adolescents (2). A study in rural China (*n* = 1,908) showed that 12.2% of adolescents met the DSM-5 diagnostic criteria for NSSI, and about 29% of them had at least one history of self-harm in the last year (3). A recent meta-analysis reported that the prevalence of self-injury among Chinese adolescents was 22.37% (4). In addition to the pain caused by NSSI, it is also the strongest predictor of suicidal behaviors (5, 6).

Previous studies have suggested that NSSI is widely present in adolescents and young adults with various mental illnesses (borderline personality disorder, depression, bipolar disorder, eating disorders, substance abuse, etc.) (7–9). For example, Kaess et al. reported that up to 60% of adolescent patients with mental illness have engaged in NSSI once in their lives, and 50% of them have repeated NSSI (10). Xiao et al. found that the comorbidity rate of depression and NSSI is very high, especially in adolescent and young adult patients (11). Longitudinal studies also suggest that depression is the most common risk factor for NSSI (2, 12). It remains unclear why some patients with depression have self-harm behavior due to the relatively high heterogeneity between the studies. Evidence supports that the complex interaction of biological factors and psychosocial factors may be a risk factor for self-harm behavior (13, 14).

Childhood traumatic experience is one of the psychosocial factors for NSSI. Childhood trauma includes physical experience, emotional experience, sexual abuse, physical and emotional neglect, and poverty (15). Studies have revealed that childhood trauma is related to NSSI in the general population, as well as in patients with mental illness (16–19). In addition, many studies have examined the relationship between different types of childhood trauma and NSSI. Paul et al. found that compared with physical abuse and sexual abuse, children whose basic physical needs are not met may face a greater risk of self-harm (20). Another study of middle-school students in China reported that childhood sexual abuse is associated with different degrees of fatal self-harm. Highly fatal self-harm is related to physical peer injury, sexual abuse, emotional abuse, and emotional neglect during childhood, and low-fatal self-harm behavior is associated with childhood peer injury, family life stress event scores, and childhood sexual abuse (21). Studies exploring the psychological mechanism between childhood trauma and NSSI demonstrate that mood regulation abnormalities and depressive symptoms are associated with a higher risk of self-harm (22, 23).

Biological risk factors of NSSI mainly focused on neuroendocrine, such as the hypothalamic–pituitary–adrenal (HPA) axis. Studies have suggested that childhood traumatic experiences may lead to abnormal HPA axis function, thereby making individuals more prone to psychopathological symptoms in adulthood (24). As the main stress response system, the HPA axis can be activated to release glucocorticoids. Prolonged activation of the HPA axis and the release of related glucocorticoids in childhood have been demonstrated to affect the brain regions related to mood regulation. The abnormal functional development of brain regions, including the hippocampus and prefrontal cortex (PFC), may in turn lead to HPA axis dysfunction (25), suggesting that childhood trauma may interact with the HPA axis and lead to emotional regulation disorders and related disorders, such as NSSI (26). Interestingly, previous studies have found that self-injured individuals had a slower salivary cortisol response after receiving the Trier Social Stress Test (TSST) (27–29), but other studies reported that NSSI individuals showed more pronounced HPA axis activation after receiving cold pain stimulation (30, 31). Another study on siblings found that salivary cortisol levels in the NSSI group significantly decreased after the trauma interview, while the hair cortisol levels in the NSSI group were significantly higher than those without NSSI (32). However, a recent study (*n* = 117) did not find a statistically significant difference in blood cortisol levels between individuals with NSSI and non-NSSI individuals. It is worth mentioning that one third of the individuals with NSSI met the diagnostic criteria for borderline personality disorder, and 62% of patients met the diagnostic criteria for depression (33).

Based on recent evidence regarding childhood trauma and abnormal cortisol levels in self-injury patients, as well as a close relationship between cortisol and childhood trauma, some studies have focused on the HPA axis function and childhood trauma experience in individuals with NSSI in the general population. So far, only one study on the HPA axis function of depression with NSSI suggests that depression patients with NSSI had lower saliva cortisol levels and different trajectories of cortisol secretion in the context of social stress compared with healthy controls and depression patients without NSSI (29). However, no study has been conducted to investigate HPA axis function (including blood cortisol) and childhood adversity in depression with or without NSSI. Therefore, the main purpose of this study is to compare whether there is a difference in childhood adversity and HPA axis function in depression patients with or without NSSI. We hypothesize that patients with major depressive disorder (MDD) and NSSI have lower HPA axis function and more severe childhood trauma. Moreover, decreased serum cortisol levels may be related to childhood trauma in patients with MDD and NSSI.

## Methods

### Subjects

This is a cross-sectional study of a representative group of inpatients with depression. From March 2020 to March 2021, a total of 324 patients with depression were admitted to the Depressive Disorders Department of Shenzhen Kangning Hospital. Of the *n* = 324 patients, only 126 depressive patients (29 males and 97 females) met all eligibility criteria of this study. The enrolment inclusion criteria were as follows: (1) depression diagnosed according to Mini-International Neuropsychiatric Interview; (2) Hamilton depression scale (HAMD17) score ≥17 (indicating moderate to severe); (3) age range from 16 to 35; (4) educational level: junior high school and above. Exclusion criteria were as follows: (1) borderline personality disorder diagnosed by the Schedule Clinical Interview for personality disorders (SCID-II); (2) combined with severe physical disease or brain organic disease; (3) receiving hormone therapy; (4) hypercortisolism in the past; (5) with psychoactive substances abuse or dependence before recruitment (except for tobacco and alcohol); (6) a history of a large quantity of alcohol abuse within 1 week before enrollment; (7) combined with other mental disorders (e.g., schizophrenia, bipolar disorder, autism, etc.).

Non-suicidal self-injury was diagnosed as the criteria stipulated by the Diagnostic and Statistical Manual of Mental Disorders, 5th Edition (DSM-5) (34). All subjects were divided into two groups according to the DSM-5 criteria for NSSI. Among them, 80 patients with depressive disorder met the diagnostic criteria of non-suicidal self-harm behavior in DSM-5, so they were in the depressive disorder with non-suicidal self-harm behavior group (MDD/NSSI group). A total of 46 patients with depressive disorder have never had any self-harm behavior before, so they were in the depressive disorder without non-suicidal self-harm behavior group (MDD group).

The ethics committee of Shenzhen Kangning Hospital approved the research protocol. All participants provided written informed consent.

### Clinical Evaluation

The 17-item Hamilton Depression Rating Scale (HAMD) was conducted to assess the severity of depression. It was translated into Chinese and evaluated the reliability and validity (35). HAM-D score level of depression: 0–7 none; 8–16 mild; ≥17 moderate to severe (36). The Beck Depression Inventory (BDI-II) was also used to assess the depressive symptoms. BDI-II has been translated into Chinese and evaluated the reliability and validity (37). The Childhood Trauma Questionnaire (CTQ) was used to screen patients for traumatic childhood experiences in childhood. CTQ has been translated into Chinese and evaluated the reliability and validity (38). All patients also completed a clinical report form which included the information of demographic data, age at first self-harm, and current/past pharmacological treatment.

### Blood Sample Collection and Testing

After an overnight fast on the second day of admission, venous blood was collected from all patients between 7 and 8 a.m. Centrifugation, serum separation, and measurement were performed within 2 h. Then, the technicians used the electrochemiluminescence method to measure the levels of cortisol on the day of the blood sampling. The instrument was a Beckman Coulter particle chemiluminescence immunoanalyzer (UniCel DxI 800) with the kits (number: YZB/USA0523-2005, Beckman Coulter Inc.). An investigator used code numbers to keep the identity of all patients blinded until cortisol levels were measured for all samples.

### Statistical Analysis

Kolmogorov–Smirnov single sample test was used to test whether serum cortisol was in a normal distribution in patients. Demographic and clinical variables of the MDD/NSSI group and the MDD group were compared by using chi-square for the categorical variables or the analysis of variance (ANOVA) for the normally distributed continuous variables or the rank-sum test for non-normal distribution variables. Next, a one-way analysis of variance for analysis of covariance (ANCOVA) was performed to compare the severity of depressive disorder between groups with sex, age, age at the first onset of depressive disorder, smoking, drinking, and the dose of antidepressants as covariates. In addition, Pearson's or Spearman's correlation was used to assessing the relationship between variables. A further binary logistics regression analysis (entry method) was carried out with self-harm as the dependent variable, depression, cortisol levels, and CTQ as independent variables, and sex, age, age at the first onset of depressive disorder, smoking, and drinking as covariates. Then, sex, age, age at the first onset of depressive disorder, smoking, and drinking were used as covariates (entry method), and statistically significant variables (*p* > 0.1) in the initial regression analysis were included in the independent variables to perform regression analysis again (backward method).

All statistical analyses used SPSS version 20.0, and the statistical significance was set at a 2-tailed *p* < 0.05.

## Results

### Clinical and Demographic Data

Table 1 shows the demographic characteristics of the MDD/NSSI group and MDD group. There was no difference in age, sex, dose, and the type of antidepressants between the MDD group and MDD/NSSI group (all *p* > 0.05). However, significant differences were observed in age of onset, smoking status, and alcohol intake between the two groups (all *p* < 0.05), which were adjusted in the subsequent analyses.

**Table 1:** Demographic and clinical characteristics of MDD patients with and without NSSI.

	**MDD (*n* = 4636.5%)**	**MDD/NSSI (*n* = 8063.5%)**	***p*-value**
Age (years)	21.70(3.7)	20.86(3.96)	0.247
Female, *n*(%)	33(71.7%)	64(80.0%)	0.289
Age onset (years)	18.91(3.62)	17.39(3.65)	0.025
Smoking status, *n*(%)	6(13.0%)	31(38.8%)	0.002
Alcohol intake, *n*(%)	9(19.6%)	46(57.5%)	0.000
Medication			
SSRI, *n*(%)	46(100%)	80(100%)	
Antipsychotic, *n*(%)	5(10.9%)	12(15.0%)	0.514
Mood stabilizer, *n*(%)	4(8.7%)	14(17.5%)	0.174
Age at first attempt (years)	NA	15.78(4.18)	
BMI (kg/m^2^)	21.28(4.79)	21.62(4.47)	0.683
BDI-II	23.46(7.43)	33.88(9.63)	<0.001

No significant relationships between the cortisol levels and age, age of onset, sex, smoking status, and alcohol intake were observed either for the entire group or when the patients with MDD and patients with MDD/NSSI were analyzed separately (all *p* > 0.05). For patients with MDD/NSSI, there was no significant association of cortisol levels with the age of onset or duration of illness (all *p* > 0.05).

### Serum Cortisol in MDD Patients With and Without NSSI

The serum cortisol levels in patients with MDD/NSSI were lower than those in patients with MDD (253.11 ± 108.75 vs. 320.71 ± 134.22 ng/ml, F = 9.486, *p* < 0.01). The difference in serum cortisol levels between MDD/NSSI and MDD groups remains significant after controlling for age, sex, age of onset, smoking status, alcohol intake, and the dose of antidepressants and BDI-II (F = 5.567; *p* < 0.05) (Table 2).

**Table 2:** Serum cortisol in MDD patients with and without NSSI.

	**MDD(*n* = 46)**	**MDD/NSSI (*n* = 80)**	**F**	***p***	**Adjusted F**	***p***
Cortisol,	320.71(134.22)	253.11(108.75)	9.486	0.003	5.567	0.020
mean (±SD)				

*Adjusted F indicates the F value controlled for age, gender, age of onset, smoking status, alcohol intake, medication and BDI-II*.

### Serum Cortisol and Psychopathology of Patients

**Figure 1:**
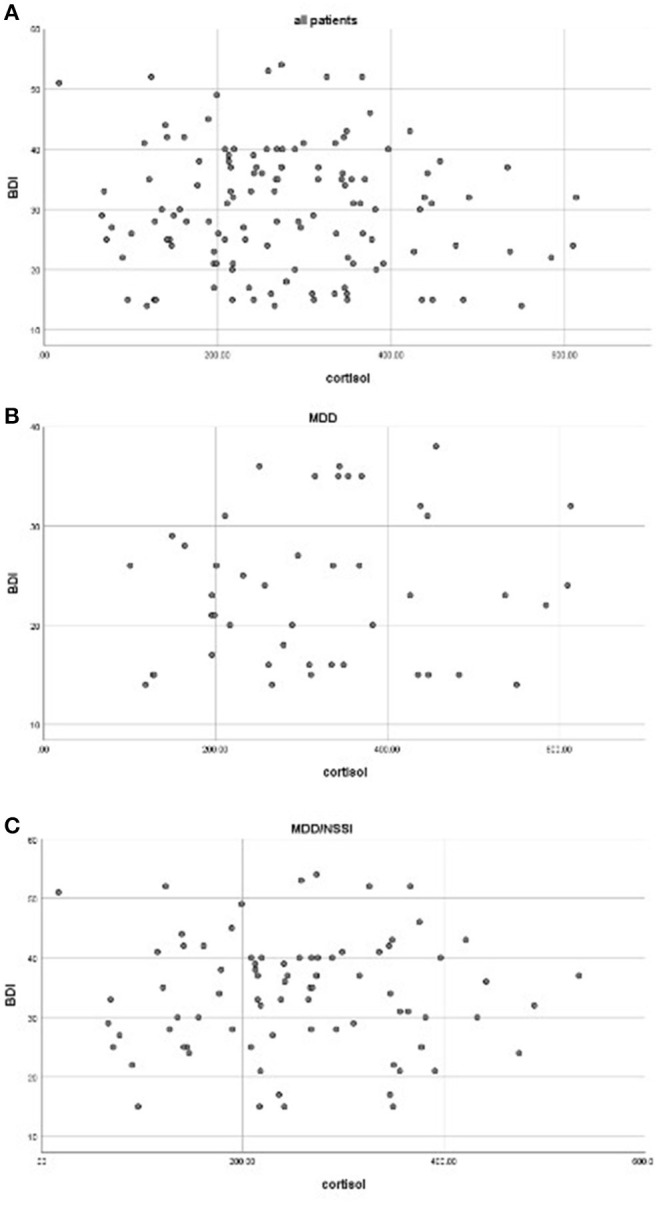
Correlations between cortisol levels and BDI scores in all patients **(A)**, MDD **(B)**, and MDD/NSSI patients **(C)** (all *p* > 0.05).

The average scores of BDI-II in the MDD/NSSI group were significantly higher than the MDD group (33.88 ± 9.63 vs. 23.46 ± 7.43, F = 40.104, *p* < 0.001) (Table 1). Correlation analyses showed no significant association between cortisol levels and the BDI score for the whole patients (*r* = −0.086, *p* = 0.339) or when the patients with MDD (*r* = 0.017, *p* = 0.880) and MDD/NSSI (*r* = 0.128, *p* = 0.396) were compared separately. The scatterplots were used to show the relationship between BDI scores and cortisol levels (Figure 1).

### Experiences of Childhood Trauma in Patients

The CTQ total score and its subscore are shown in Table 3. Patients with MDD/NSSI had significantly higher CTQ total and its four subscores than patients with MDD (all *p* < 0.05), except for the physical neglect.

**Table 3:** Experiences of childhood trauma between MDD patients with and without NSSI.

	**MDD**	**MDD/NSSI**	**F/Z**	***p***
Physical abuse, mean (SD)^*^	6.85(3.13)	9.14(5.04)	−2.716	0.007
Emotional abuse, mean (SD)	10.15(4.99)	13.35(5.03)	11.870	0.001
Sexual abuse, mean (SD)^*^	5.41(1.15)	6.24(2.50)	−2.180	0.029
Physical neglect, mean (SD)	9.33(3.60)	10.60(4.14)	3.027	0.084
Emotional neglect, mean (SD)	12.91(5.50)	16.95(4.26)	21.113	<0.001
Total score, mean (SD)	44.76(15.19)	56.29(14.43)	−4.256	<0.001

* *Indicated using Mann-Whitney Test*.

In addition, correlation analysis was used to examine the association between the depressive symptoms and childhood adversity in all patients or the patients with MDD and MDD/NSSI separately. For all patients with MDD, we found significant positive associations between BDI-II scores and CTQ total and its four subscores (all *p* < 0.05), except for the sexual abuse. For MDD/NSSI patients, positive associations between BDI-II scores and CTQ total score, emotional abuse, and emotional neglect subscores were observed (Table 4). For MDD patients without NSSI, no correlation was found between BDI-II scores and total CTQ score or any subscale scores.

**Table 4:** Correlations between BDI and childhood trauma in all patients and MDD/NSSI patients.

	***r* (all patients)**	***p***	***r* (MDD/NSSI)**	***p***	***r* (MDD)**	***p***
Physical abuse^*^	0.223	0.012	0.042	0.714	0.276	0.068
Emotional abuse	0.333	<0.001	0.328	0.003	−0.008	0.958
Sexual abuse	0.070	0.434	−0.037	0.747	0.027	0.859
Physical neglect	0.217	0.015	0.199	0.077	0.074	0.625
Emotional neglect	0.298	0.001	0.256	0.022	−0.065	0.666
Total	0.343	<0.001	0.286	0.010	0.042	0.783

* *Indicated using spearman collection analysis*.

### Correlation Between Cortisol and Experiences of Childhood Trauma

Correlation analyses revealed negative associations between the levels of cortisol and the CTQ total score, emotional abuse, and emotional neglect subscores for all patients (all *p* < 0.05). For patients with MDD/NSSI, there is only a negative association between the level of cortisol and the emotional neglect subscore (*p* < 0.05) (Table 5). For MDD patients, no correlation was found between cortisol levels and total CTQ score or any subscale scores.

**Table 5:** Correlations between cortisol and childhood trauma in all patients and MDD/NSSI patients.

	***r* (all patients)**	***p***	***r* (MDD/NSSI)**	***p***	***r* (MDD)**	***p***
Physical abuse^*^	−0.056	0.533	0.006	0.960	−0.022	0.883
Emotional abuse	−0.276	0.002	−0.220	0.050	−0.210	0.161
Sexual abuse	−0.020	0.827	0.030	0.293	0.050	0.743
Physical neglect	−0.137	0.126	−0.082	0.472	−0.136	0.368
Emotional neglect	−0.183	0.040	−0.068	0.551	−0.118	0.436
Total	−0.209	0.019	−0.119	0.292	−0.137	0.363

* *Indicated using spearman collection analysis*.

Next, logistic regression model 1 was performed to investigate the correlation of childhood adversity with NSSI after adjusting for age, sex, smoking, and alcohol intake. The results showed that BDI-II, low cortisol levels, physical abuse, emotional abuse, sexual abuse, and emotional neglect were associated with NSSI (all *p* < 0.05) (Table 6). Then, all the variables obtained in model 1 that were significantly correlated with NSSI were included in regression model 2 for further analysis using the backward LR method after adjusting for age, sex, smoking status, and alcohol intake. We found that low cortisol levels (OR = 0.996, *p* = 0.042), BDI-II scores (OR=0.1.155, *p* < 0.001), and emotional neglect subscore (OR = 1.134, *p* = 0.022) were risk factors for NSSI.

**Table 6:** Logistic regression of the variables with NSSI.

	**Model 1 (OR, 95%CI)**	***p***	**Model 2 (OR, 95%CI)**	***p***
Cortisol	0.996(0.993–1.000)	0.037	0.996(0.992–1.000)	0.042
BDI-II	1.146(1.080–1.216)	0.000	1.155(1.081–1.234)	0.000
Physical abuse	1.125(0.998–1.268)	0.054	NA	
Emotional abuse	1.086(0.991–1.191)	0.078	NA	
Sexual abuse	1.295(0.965–1.736)	0.085	NA	
Physical neglect	1.027(0.921–1.145)	0.631	NA	
Emotional neglect	1.152(1.051–1.262)	0.003	1.134(1.019–1.263)	0.022

*Logistic regression model 1 was performed to investigate the correlation of individual variable with NSSI after adjusting for age, sex, life-style factors like smoking, alcohol intaking. Logistic regression model 2 was performed including all the variables which were significantly associated with NSSI in model 1 (p < 0.1) using Backward LR method after adjusting for age, sex, life-style factors like smoking, alcohol intaking (p < 0.05)*.

## Discussion

In this study, we found that (1) patients with MDD/NSSI experienced more childhood trauma and severe depressive symptoms than MDD patients without NSSI. (2) Cortisol levels were lower in patients with MDD/NSSI. (3) The levels of cortisol were negatively associated with emotional neglect only in patients with MDD/NSSI, rather than in MDD patients without NSSI. (4) Low cortisol levels, the severity of depressive symptoms, and emotional neglect were risk factors for NSSI in young patients with MDD.

The current study found that the blood cortisol levels of MDD/NSSI patients were significantly lower than those of MDD patients without NSSI. Previous studies also found that both non-clinical individuals with NSSI (27, 28) and patients with MDD/NSSI (29) had lower cortisol levels than non-NSSI individuals under stress, indicating that NSSI was related to HPA axis function. However, the other two studies did not find the differences in cortisol levels between NSSI and non-NSSI individuals in a non-stimulated background (39, 40). The discrepant findings may be explained by many factors, such as samples (blood, saliva, or urine), disease status (first-episode or chronic), the severity of illness, age of onset, duration of illness, etc. Cortisol is a hormone secreted by the adrenal cortex and is regulated by the central hypothalamus and pituitary. Therefore, to a certain extent, the levels of cortisol reflect the function of the HPA axis. Our study found that the levels of cortisol in patients with MDD/NSSI were lower, suggesting that HPA axis alteration may be involved in the pathogenesis of NSSI in patients with depression.

Another finding of this study was that patients with MDD/NSSI had significantly higher BDI-II scores than the MDD group, which is consistent with previous studies on individuals with NSSI in the community (5, 11, 41, 42). These findings suggest that individuals with NSSI are more depressed than individuals without NSSI, and patients with severe depression may be more prone to NSSI. We also found that the MDD/NSSI group's CTQ total score and emotional abuse, physical abuse, sexual abuse, and emotional neglect scores were significantly higher than the MDD group. In addition, BDI-II scores were positively correlated with the scores of trauma, emotional abuse, physical abuse, emotional neglect, and physical neglect in patients with MDD, which is consistent with previous studies (43–45). However, none of the previous studies mentioned whether the subjects were comorbid with NSSI. Our further separate analysis of the MDD and MDD/NSSI groups indicates that the positive correlation between the score of depressive symptoms and the total CTQ score, emotional abuse, and emotional neglect only exists in the MDD/NSSI group, but not in MDD. Differences in the association of the severity of depressive symptoms with CTQ score suggest that NSSI is more closely related to childhood traumatic experiences than depression. Further regression analysis found that emotional neglect may be a risk factor for NSSI, which indicates that insufficient emotional care for children during childhood parenting may make them more likely to engage in self-harm behavior in young adults.

The current study further found that blood cortisol levels were negatively correlated with total CTQ score, emotional abuse, and emotional neglect subscores. In the MDD/NSSI group, blood cortisol levels were negatively correlated with emotional neglect, but in the MDD group, cortisol levels were not correlated with CTQ scores. A previous study has found that blood cortisol levels were different between patients with MDD with childhood trauma and those without childhood trauma. Moreover, the salivary cortisol levels were positively correlated with childhood trauma experience (39). However, the findings from Liu were inconsistent with our findings. It is worth mentioning that Liu did not determine whether patients with depression were comorbid with NSSI. Some studies have found that the individuals with NSSI from the community populations had HPA axis dysfunction, suggesting that the relationship between cortisol levels and childhood trauma experiences in patients with MDD may be affected by NSSI.

The current study has several limitations. First, this study only recruited patients with depression and did not include healthy control subjects, so it is unclear whether the findings of HPA axis abnormality are generalized to other populations. Considering that NSSI is currently diagnosed by DSM-5 as an independent disease, future studies should include broader recruitment criteria and comparisons in the general population. Second, the MDD/NSSI group did not match the MDD group in the demographic data such as the age of onset of depression, smoking, drinking, and severity of depression, which may potentially affect the serum cortisol levels. Although we have adjusted these confounding factors in the statistical analysis, it may not be possible to completely avoid the bias caused by the imbalance of these variables. In addition, the current study is only a cross-sectional study, so it is impossible to derive a causal relationship. Future longitudinal studies are warranted to clarify the relationship between childhood traumatic experiences, non-suicidal self-injury behaviors, and abnormal HPA axis function. Third, this study is limited by the small sample size. We used the G^*^Power 3.1.9.2 program to calculate the sample size based on effect size and found that the sample size should be 278 to obtain a power of 0.8 at α < 0.05 (two-tailed). Thus, 126 patients in the present study were insufficient and the negative results may be due to the lower power.

In summary, this study found that patients with depression and non-suicidal self-harm behavior had significantly lower blood cortisol levels than those without self-harm behaviors. Lower cortisol levels may be a risk factor for non-suicidal self-harm behaviors in patients with depression, suggesting that abnormal cortisol levels may play a role in the pathogenesis of non-suicidal self-harm behavior in patients. In addition, MDD patients with non-suicidal self-harm had more severe childhood trauma experiences and depressive symptoms than those without non-suicidal self-harm behavior. Childhood emotional neglect and depression are risk factors for self-harm behavior in patients with MDD, and patients with severe depression may be more prone to non-suicidal self-harming behaviors. This finding remains preliminary due to the limited sample size and needs replication in a large sample.

## Data Availability Statement

The raw data supporting the conclusions of this article will be made available by the authors, without undue reservation.

## Ethics Statement

The studies involving human participants were reviewed and approved by the Ethics Committee of Shenzhen Kangning Hospital approved the research protocol. All participants provided written informed consent. The patients/participants provided their written informed consent to participate in this study.

## Author Contributions

MX, YZ, and BP were responsible for study design, statistical analysis, and manuscript preparation. BP, HL, GC, HF, and JL were responsible for recruiting the patients, performing the clinical rating, and collecting the clinical data. MX, YZ, and HF were evolving the ideas and editing the manuscript. MX and BP were involved in writing the protocol and cowrote the paper. All authors have contributed to and have approved the final manuscript.

## Funding

This study was supported by Shenzhen Key Medical Discipline Construction Fund (No. SZXK043) and Shenzhen Fund for Guangdong Provincial High-level Clinical Key Specialties (No. SZGSP013). These sources have no further role in the study design, data collection and analysis, report writing, and decision to submit the paper for publication.

## Conflict of Interest

The authors declare that the research was conducted in the absence of any commercial or financial relationships that could be construed as a potential conflict of interest.

## Publisher's Note

All claims expressed in this article are solely those of the authors and do not necessarily represent those of their affiliated organizations, or those of the publisher, the editors and the reviewers. Any product that may be evaluated in this article, or claim that may be made by its manufacturer, is not guaranteed or endorsed by the publisher.
